# Development of an Intervention to Support the Reproductive Health of Cambodian Women Who Seek Medical Abortion: Research Protocol

**DOI:** 10.2196/17779

**Published:** 2020-07-10

**Authors:** Elisa Oreglia, Sokhey Ly, Camille Tijamo, Amra Ou, Caroline Free, Chris Smith

**Affiliations:** 1 Kings College London London United Kingdom; 2 Marie Stopes International Cambodia Phnom Penh Cambodia; 3 London School of Hygiene and Tropical Medicine London United Kingdom; 4 School of Tropical Medicine and Global Health Nagasaki University Nagasaki Japan

**Keywords:** reproductive health, abortion, Cambodia, mHealth, mobile phone

## Abstract

**Background:**

In Cambodia, abortion has been legally permitted on request during the first trimester of pregnancy since 1997. However, although there has been an increase in the percentage of women having induced abortion and medical abortion, there has also been a decrease in the percentage of women who say they received help from a health worker with their abortion. These data point toward the *demedicalization* of abortion, and although medical abortion has been shown to be safe, there are concerns about safety, given the variety of available products and counseling provided. These concerns are particularly relevant for female factory workers, who typically come from rural areas where access to good health care and information about reproductive health care is limited.

**Objective:**

This study aims to understand the reproductive health needs of female Cambodian garment factory workers after medical abortion from a multidisciplinary and mixed-methods perspective, focusing on how they seek and share medical abortion- and health-related information; how they use their mobile phones for this and other purposes; what cultural challenges exist around reproductive health; and how they might be magnified or mitigated by mobile phones, linguistic challenges around health care, and mobile phone use. The main purpose of this study is to combine multidisciplinary methods, theories, and expertise to gain new, culturally grounded insights into family planning and medical abortion in Cambodia, but the findings could help inform the development of a relevant intervention to support comprehensive postabortion care.

**Methods:**

The methods proposed are interviews and participant observation among factory workers, health providers, and mobile phone providers; a linguistic analysis of relevant data (interview transcripts, web-based sources, and other fieldwork materials); and digital methods to understand what kind of information about medical abortion exists on the web in Cambodia and how it is accessed by the targeted population.

**Results:**

The data collection part of the project will end on December 31, 2020. The team conducted 67 semistructured interviews with female factory workers, women who sought a medical abortion, health providers, and mobile phone providers; participant observation with factory workers and health providers; and an analysis of YouTube and Facebook to understand what kind of information is available, who creates it, and how it is used. The team is currently performing data analysis, and the findings are clustered around (1) the use of mobile phones and digital resources for health-related and medical abortion-related information, (2) the experience of medical abortion care, and (3) the development of an intervention through edutainment videos.

**Conclusions:**

The project highlights both the widely untapped potential of using digital platforms (especially YouTube and Facebook) to distribute accurate information on medical abortion and the challenges in providing individual information via mobile phones while respecting individuals’ privacy.

**International Registered Report Identifier (IRRID):**

DERR1-10.2196/17779

## Introduction

In Cambodia, abortion has been legally available on request during the first trimester of pregnancy since 1997 [1,2]. Drugs used in medical abortion containing mifepristone and misoprostol were approved in 2010 and made available in a restricted number of health facilities [2,3]. Although nationwide surveys report an increase in the percentage of women having induced abortion and medical abortion, they also report a decrease in the percentage of women who say they received help from a health worker with their abortion. These data point toward the *demedicalization* of abortion, a phenomenon that is being observed globally, as evidenced by the increased use of medical abortion pills at home with limited contact with trained health care professionals [4]. Although medical abortion has been shown to be safe and up to 98% effective for early pregnancy terminations, there are concerns about safety, given the variety of available products and counseling provided [5]. Further research on this topic is needed.

The Cambodian garment industry accounts for a significant proportion of formal employment of the female labor force and is an important contributor to Cambodia’s economy. As of 2015, there were 837 garment factories in Cambodia, providing formal employment to approximately 700,000 garment factory workers, of which approximately 85% are women, most of whom are of reproductive age [6,7]. It has been estimated that one-fourth of all Cambodian women between 18 and 29 years are working in garment factories. Typically, these women come from rural areas where access to high-quality information about reproductive health is limited. Furthermore, it is also likely that migrating to urban areas puts women in a disadvantaged position as they are no longer able to receive family or community support and are more vulnerable to sexual exploitation, often resulting in unwanted pregnancies and sexually transmitted infections [6].

Marie Stopes International Cambodia (MSIC) is part of the Marie Stopes International (MSI) global partnership. MSIC has been delivering high-quality sexual and reproductive health services in Cambodia since 1998, covering seven provinces. MSIC provides family planning, contraception, safe abortion, and postabortion care within the legal framework of Cambodia. In addition, MSIC operates a contact center that serves as a link between MSIC and the general public for all the services that MSIC provides and for general information about sexual and reproductive health, including family planning, safe abortion, postabortion care, and other sexual and reproductive health issues. MSIC has strong relationships with garment factories having undertaken 2 relevant projects in recent years: Partnering to Save Lives (PSL) and Worker Health. PSL is a project funded by the Australian Department of Foreign Affairs and Trade and is a collaboration between MSIC, CARE Cambodia, and Save the Children that started in 2013. PSL conducted a baseline survey of the knowledge, attitudes, and practices of garment factory workers in 2014 and 2016, which provides background information about our research participants in terms of mobile phone ownership, awareness of abortion services, and contraception options [7]. Worker health is a project funded by the United States Agency for International Development and a collaboration between MSIC and the Population Council to increase the access of garment factory workers to and utilization of health services, particularly high-quality reproductive health and voluntary family planning services. Their systematic review of garment sector health interventions showed that there was limited use of mobile health services [6].

This project, a collaboration between the London School of Hygiene and Tropical Medicine (LSHTM), the School of Oriental and African Studies (SOAS) University of London, King’s College London, and MSIC, funded by the United Kingdom Arts and Humanities Research Council (AHRC), aims to complement previous and ongoing projects but focuses particularly on support for reproductive health after medical abortion and health-seeking behaviors in general. Understanding the reproductive health needs of female Cambodian garment factory workers after medical abortion, together with understanding how they seek and share information and how they use mobile phones, could help inform the development of a relevant intervention to support comprehensive postabortion care. To do so, however, it is necessary to understand (1) how they use mobile phones and how they fit within offline practices of information sharing; (2) the cultural challenges around reproductive health and how they might be magnified or mitigated by mobile phones; and (3) the linguistic challenges around mobile phone use, for example, language input, or oral versus written practices.

Women seeking medical abortion from clinics such as MSI are routinely offered a follow-up appointment, but many will not attend unless they have a problem. For those seeking medical abortion from informal clinics and pharmacies, very little is known about outcomes such as follow-up rates, use of contraception, repeat abortion, and complications (such as uterine rupture or incomplete abortion), and observational studies report that women are often given little information on how to use medications and postabortion family planning [2]. Against this background, mobile phone communication is a potential way to maintain communication with women after they have obtained medical abortion drugs from a health care provider by overcoming distance and access barriers and potentially providing new modes of interaction, such as synchronous and asynchronous voice communications through instant messaging apps. There is an emerging body of evidence supporting the feasibility, acceptability, and efficacy of digital interventions for behavior change [8,9]. However, as smartphones become increasingly common in low- and middle-income countries, a new generation of digital health apps is being developed without considering the populations they target from a sociocultural and linguistic perspective. This is the gap that the collaboration between LSHTM, SOAS, and Marie Stopes International addresses: bringing health care, sociocultural, linguistic, and local expertise together to understand how mobile phones, existing communication patterns, and health care interventions can come together in a way that is cost- and time-effective for the providers and fit into the cultural patterns of language, information exchange, and social interactions of the targeted population.

This study aims to conduct a joint research project using qualitative methods from a multidisciplinary perspective to, first, study health information gathering and sharing among factory workers and reproductive health issues after medical abortion in Cambodia from medical, cultural, and information-sharing practice perspectives; and second, design a pilot intervention delivered by mobile phone to support the reproductive health of women who work in factories around Phnom Penh.

## Methods

### Methodological Approach

This study will focus on the following populations: first, women working in selected garment factories around Phnom Penh; second, private health care providers near selected garment factories; and third, women working in garment factories seeking medical abortion from private providers near garment factories. Women working in garment factories will be selected if they are willing to be interviewed and observed at work and in their living quarters, own or do not own a mobile phone, are using contraception or have used medical abortion pills or not, and are 18 years and older. Garment factories will be selected if they are located near Phnom Penh and have an existing relationship with MSIC. Private providers will be selected based on whether they are a pharmacy or small private clinic, are located near Phnom Penh, are selling medical abortion pills (not limited to registered products), the manager and at least one worker is willing to participate, and are employing workers aged 18 years or older. Women seeking medical abortion from private providers will be invited to participate in the study based on if they (1) are using medical abortion pills, purchased from study pharmacies during the study period; (2) purchased medication by themselves; (3) had access to a mobile phone and were willing to be followed up by phone with questions about their abortion periodically after taking the first drug; (4) are 18 years and older; (5) if their gestational age is less than 9 weeks (calculated from the last menstrual period), have a confirmed pregnancy (self-reported); and (6) are willing and able to give informed consent.

The research project in Cambodia will draw on qualitative methods from public health and the humanities/social sciences. One of the goals will be to use more ethnographic perspectives within the context of the intervention mapping (IM) approach commonly used to develop health behavior change interventions. Ethnographic work will be employed to reach a holistic picture of the environment in which the women live and work, without focusing exclusively or necessarily on reproductive health care. Ethnographic work will take place at the very beginning of the project as an exercise in idea generation and contextualization for the subsequent phase, IM. IM is a well-established methodology for conducting interdisciplinary intervention development and is flexible enough to be adapted to different cultural settings [10]. It will allow the research partnership to build on the IM approach recently used by LSHTM colleagues (Ona McCarthy/CF) to develop an intervention to support contraception use in Palestine, Bolivia, and Tajikistan [11] as well as qualitative methods used by the SOAS researchers, methods that require constant refining of research questions [12], and user-centered design principles [13]. IM is a cumulative process that often necessitates moving back and forth through the following steps: (1) needs assessment, (2) specifying behavior change resulting from the intervention, (3) designing the intervention, (4) producing and refining the intervention content, (5) planning intervention implementation, and (6) planning intervention evaluation. This project will focus on steps 1-4 and will bring a cultural and sociological perspective to these phases. The methodological collaboration will focus, in particular, on the needs assessment and intervention design phases. A key aspect of the partnership will be to explore and discuss findings in relation to the theories and perspectives of each of the disciplines contributing to it. The following research activities are planned, with activities 1 to 3 focusing on IM step 1: (1) participant observation and interviews regarding the current behaviors of women in health information gathering and sharing, (2) interviews with private providers and garment factory infirmary workers in the vicinity of garment factories that provide or refer for medical abortion, (3) interviews with women seeking medical abortion from private providers, (4) specifying behavior change to result from the intervention, (5) designing the intervention, and (6) producing and refining intervention content.

### Activity 1: Participant Observation and Interviews Regarding Women’s Current Behaviors in Health Information Gathering and Sharing

The objectives of activity 1 are to understand how women look for and share information related to health care, in particular, abortion and postabortion services, in their everyday life; to understand the local context, in terms of resources, access, and geography, and to generate ideas for activity 2. Field research will begin with observations, *hanging out*, and semistructured interviews among female factory workers in the targeted neighborhood. To gain a holistic understanding of *needs* beyond specific health care–related ones and thus discover what kind of information channels they use, and who they trust, the team will try to recruit a larger pool of participants than those who will participate in activity 2 (eg, 25-50 participants). Researchers will conduct participant observation in key places such as garment factories with links to MSIC (eg, during lunch breaks and before and after work), garment factory infirmaries, pharmacies, and other places where young women meet in their leisure time. Locations and potential participants will be identified with the help of the project’s Cambodian staff according to the criteria discussed earlier. Fieldwork will be carried out by the main principal investigators (PIs) and UK-based students with Khmer language skills and by a Cambodian research assistant to ensure that it is done in a culturally sensitive manner.

A possible plan for this initial fieldwork is as follows. The researchers will live in a guesthouse in the area to experience the environment and familiarize themselves with life in the neighborhood (eg, mapping out health care–related resources, including restaurants, shops, and other points of congregation; experiencing life inside and outside the factories and where women live; etc). On the first day, they will go to the factories that have agreed to participate in the project and be introduced to the staff and potential research participants (Multimedia Appendix 1). The first week will be spent *hanging out* at both the factory and outside, befriending different potential participants, having meals with them, and, if they agree, joining them for activities outside the factory or to visit their living quarters. The researchers will explain the purpose of their research and ask for permission to take photos and record conversations if appropriate (Multimedia Appendix 2). Alongside participant observation and hanging out with women, researchers will conduct participant observation in local pharmacies and a clinic. With the consent of the participants, the researchers will follow a nurse or an administrative person for a day to see who they interact with; what kind of questions they ask; and how they use computers, phones, and mobile phones. When there are close interactions with the clinic’s patients, our role and project will be explained, and researchers will stay only with the patient’s consent. Similarly, participant observation in a private provider will include one of the researchers spending a few hours over different days in the pharmacy, observing the flux of people, and how they interact with providers to ask for health care–related information. Halfway through this first period of fieldwork, the researchers will identify among their contacts key informants to interview. The interviews will start with a general overview of informants’ use of mobile phones and then focus on health care needs and information-seeking tactics (Multimedia Appendix 3). If and when appropriate, researchers will also ask interviewees to show (rather than only describe) how they look for health-related information on their mobile phones. This phase will be followed by virtual observation of websites and specific social network pages mentioned by interviewees [14]. If invited, the researchers will also follow interviewees on social media or *friend* them on instant messaging programs or social networks. Due to privacy concerns, no material or direct quotes from these specific sources will be used in a way that could make the research participants identifiable. For example, if screenshots are captured, they will be transcribed, stripped of any identifying element, and then translated. The translation will not be used verbatim in any publication or public presentation. If quotes or images or screenshots are shown or published, they will be a composite of source material (ethical procedures are given in the *Ethical Considerations and Research Governance* section. The field site will remain the same for the duration of the project, and the UK-based and/or Cambodia-based researchers might go back at regular intervals to conduct follow-up observations and interviews.

Following feedback from reviewers from a medical background, who asked for clarifications about observation and interaction-based research methods that are common in the social sciences but interpreted differently in the medical sciences, the PIs added a clarification on how they were planning to conduct fieldwork. This is added here to the original research protocol, as it highlights that one of the challenges of conducting multidisciplinary research is that different disciplines interpret differently what on the surface are the same methods (eg, interviews and participant observation). The specific feedback asked for a detailed form for guiding observations and questioned the idea of *hanging out* and *befriending participants on social media* as potentially deceptive forms of research.

There is no specific form for guiding or recording observations. As is common in ethnographic research [15], each field researcher has a personal way of keeping a field diary, which ranges from taking notes on paper or her mobile phone during the day (or simply memorizing specific moments), then writing them up in a diary form in the evening, to taking photos of events or places that seem important, to sketching, and making voice memos. The point to stress is that the researchers are not entering the field with preconceived ideas about what is important and should be noticed and what is not. Activity 1 is ethnographic, so it is aimed at gathering a holistic picture of the environment in which the women work and live, their relationships, and the places they visit. The interviews will be semistructured, not structured (characterized by a fixed set of questions), to allow interviewers to follow up on the interviewees’ responses and discover themes that cannot be planned beforehand. The goal is to touch on the themes described in the protocol but in as open a way as possible, so that the researchers can follow the lead of the women rather than vice versa. In other words, instead of asking, “how do you use your mobile phone to look for health-related information,” which implies that the interviewee *has* used her mobile phone to look for health-related information, the interviewer would follow up on the interviewee’s description of her daily life to find out more about both mobile phone use and health-related information.

Researchers’ questions and follow-up questions will not stray outside the broad headings outlined in the topic guides, but researchers will let interviewees lead the interview and talk about any topic they would like to discuss. A relationship of mutual trust between researchers and research participants is an essential element of good ethnographic research, and it is what leads to findings of what really matters to research participants, rather than what researchers think matters**.** Similarly, the researchers will not ask to follow factory workers on social media but will accept any invitation to *friend* that is offered them, asking specifically if anonymized data can be used for data analysis. The researchers are not looking for specific information about specific individuals or screenshots of specific conversations, but there might be important and useful information about how people communicate and engage on social media that would not be otherwise accessible. Thus, the data gathered from engagement in social media will be used for internal analysis. If a screenshot is deemed a particularly useful visual support of points we discover, the researchers might ask for permission from the person or group in the conversation to use it, appropriately anonymized, in presentations and publications. Befriending research participants on social media is by now a fairly standard research method in digital anthropology [16], and the participants are always free to defriend the researcher if they do not want to be involved with them any longer. Research participants who use social media are typically very fluent in the etiquette of friending, defriending, and managing different identities and different social circles on the web. In general, in ethnographic research, there are no findings without a relationship of trust, which is built through time and interactions. This can potentially create ethical dilemmas regarding friendships that can arise between researchers and research participants [17], but they are not about the researcher disguising or lying about her role, and they are part of the constant negotiation of being in the field, carrying out research in naturalistic rather than laboratory settings. The researchers follow the recommendations from the Association of Internet Researchers Ethics Working Committee in 2012 that highlight that “ethical decision-making is best approached through the application of practical judgment attentive to the specific context” [18].

### Activity 2: Interviews With Private Providers and Garment Factory Infirmary Workers in the Vicinity of Garment Factories That Provide or Refer to Medical Abortion

The objectives of activity 2 are to understand the perspective of health care providers, such as private providers and garment factory infirmary staff, to find and share health care information, and to conduct a needs assessment. Semistructured interviews will be conducted with the staff of private providers (including pharmacies) and garment factory infirmaries where participant observation has been previously conducted to understand their perspective on how women find, share, and act on health care–related and abortion-related information. Researchers aim to interview a wide variety of stakeholders in the process as much as possible (approximately 10-20 participants), not only health care providers such as physicians and nurses but also administrative and support staff. The interviews will focus on their direct experience of being asked about health care–related and abortion-related information in person and via other means (eg, by phone) as well as their observations on the topic. Interviewers will ask questions about the characteristics of providers and staff, including age, sex, education level, and the number of years worked, their current medical abortion, and emergency contraception sales. When possible, interviewers will attempt to ground their responses using specific artifacts such as web pages, mobile phones, and anything else that suggests itself as a potential productive avenue of inquiry from the preceding participant observation and interviews. Private provider managers will be approached by a research assistant who will explain the study and inform them of the tasks that will be required by participating staff. If private provider managers consent to participate, they will be asked to sign an informed consent form. All workers at the participating provider will then be approached and asked for informed consent to participate in the study (the *Ethical Considerations and Research Governance* section provides further details).

### Activity 3: Interviews With Women Seeking Medical Abortion Services From Private Providers

The objectives of activity 3 are to increase the understanding of the local context, reasons for abortion, postabortion care issues, and mobile phone use; to increase the understanding about the feasibility of recruitment of women seeking medical abortion from pharmacies to phone (digital health) follow-up support and research studies; and to seek views on possible intervention(s). An exploratory qualitative study of approximately 10 to 20 semistructured interviews and follow-up with women seeking medical abortion will be conducted by a member of the research team. Participants will be recruited by working together with private providers. The *Ethical Considerations and Research Governance* section provides more details on the recruitment and consent procedures. Participants will be offered an initial face-to-face interview at the location of their choice, but because of potential privacy and confidentiality issues, the interviews will be conducted by phone if they prefer. Ideally, a short initial interview will be conducted at the time when the woman attends the private provider. If the participant consents, additional short interviews will be undertaken at 2 and 4 weeks to learn more about the women’s experience over the initial postabortion period. These interviews will also be conducted at the location of the participants’ choice, either face-to-face or by phone. Interviews will be recorded and transcribed if possible and with consent. In addition, there may be additional communication by mobile phone between the participants and Cambodian researchers. This could take the form of the researcher asking, “how are you?” or “if you have any problems, please call me.” No messages will contain terms related to abortion, contraception, or MSI. The aim of this communication is to document further any postabortion care issues and provide support where appropriate. Phone communications will be anonymized and documented.

### Activity 4: Specifying Behavior Change Resulting From the Intervention

The objective of activity 4 is to analyze and discuss the findings of activities 1 to 3 to specify behavior changes resulting from the intervention. The study population will be described in terms of sociodemographic characteristics using descriptive statistics. With the interviewees’ permission, interviews will be recorded and transcribed and then translated into English, and a thematic analysis undertaken. A coding frame will be developed iteratively to analyze the first few transcripts and will then be used to code all transcripts. The findings from activities 1 to 3 will be summarized and synthesized and discussed in research seminars with a range of stakeholders in Cambodia and the United Kingdom to specify potential behavior changes resulting from the intervention. The goal is to create a map of different types of communication patterns and personas that correspond to each behavior. Personas are frequently used in human-computer interaction research [19] and are an example of the type of methodological cross-pollination the project aims to create.

### Activity 5: Designing the Intervention

Activity 5 aims to seek views on possible interventions; to co-design a potential intervention with input from garment factory workers, medical abortion clients, pharmacists, MSIC research and marketing team, call center, and external organizations as required; and to develop specific message content, tone, and style. This activity builds on outputs from activity 4. The intervention components will be developed based on up-to-date empirical evidence and in collaboration with service users and providers. For the intervention design, the investigators will choose theory-informed behavior change methods to include in the intervention and deciding how to deliver them, as common in IM mapping approaches, but drawing from theories in the health field and cultural/information studies, to find the best fit with local circumstances, language, and effective interventions for supporting reproductive health after medical abortion. Although focus groups can be useful for generating ideas among participants for intervention development, this could be challenging for our target population of factory workers seeking medical abortion from private pharmacies in terms of logistics and privacy concerns.

It is anticipated that service users and health care providers will be recruited to participate in this part of the study in the same way as with activities 2 and 3. The researchers plan to undertake approximately 10 to 20 interviews with service users and providers. The study team will provide participants with information regarding the aim of the study and obtain consent as per activities 2 and 3. The researcher will elicit participants’ preferences for the intervention and seek comments from them on any preliminary messages/content developed, specifically asking about the acceptability, comprehensibility, and appropriateness of the messages/content developed and suggestions for improvement. According to the feedback received, messages will be retained, discarded, or modified, and retested, if necessary. If it is felt that an intervention delivered by a mobile phone is feasible, the research team will simultaneously evaluate digital health communication software options to deliver the intervention (eg, voice or instant messaging) with an appropriate design and level of technology with or without creative content providers. The researchers will ensure that any intervention content is consistent with the Cambodian Ministry of Health guidance.

### Activity 6: Producing and Refining Intervention Content

The objectives of activity 6 are to further seek service users’ views on acceptability, comprehensibility, and appropriateness of the messages developed and suggestions for improvement. In particular, the aims are to evaluate the reliability of technology, comprehensibility, and acceptability of message content; to produce and refine the intervention content; and to describe and publish potential intervention(s) to be evaluated. This will build further on activity 6 by testing actual messages with participants. The intervention will be tested and modified with input from approximately 10 to 20 clients identified by the study team. It is anticipated that service users and health care providers will be recruited to participate in this part of the study in the same way as with activities 2 and 3. Information for participants and consent forms will be adapted accordingly. If a digital health intervention is developed, participants will be sent intervention content to their mobile phones. The researchers will seek comments regarding the messages and suggestions regarding how messages could be improved via a subsequent phone call. The participants’ responses will inform the final modifications to the intervention.

### Ethical Considerations and Research Governance

Ethical considerations, including informed consent procedures, measures to protect confidentiality, and potential risks and benefits to participants, are considered by participant type, as follows. For all participants, the PIs will be responsible for the overall informed consent procedures for the project. CS undertook the Introduction to Good Clinical Practice course in May 2012 and has experience in recruiting women seeking abortion services for the Mobile Technology for Improved Family Planning study in Cambodia. EO has experience in undertaking ethnographic research with garment factory workers in China and completed the Ethics in Social and Behavioral Sciences course at SOAS in January 2016. They will ensure that all researchers working on the project comply with the consent procedures outlined in the protocol. All study participants (provider managers, providers, and medical abortion clients) will be informed about the purpose of the study and the potential risks and benefits relating to their participation in the study. Participants will be 18 years and older and interviewed or engaged by researchers based in the United Kingdom and Cambodia. All the study participants have the right to choose to partake in the study or refuse to participate. Study staff will be instructed to record all refusals and to attest to obtaining informed consent from the study participants. Further details of the consent procedures are described according to the type of research participant.

In Cambodian abortion law, under certain circumstances, parental consent for an abortion is required if the woman is less than 18 years of age. Therefore, conducting research on unintended pregnancy potentially raises issues that require additional safeguards beyond the scope of the design of this research project. For example, women may feel uncomfortable to consent to participate and give honest answers if they are younger than 18 years. The researchers accept the limitation that the findings of the study will not be generalizable to women younger than 18 years. In practice, the PIs do not feel that it is appropriate to ask for proof of age. They will ask women how old they are as part of the provision of information and consent to participate in the project. If they report that they are less than 18 years of age, they will be informed that they will not be able to participate in this research project. However, they will be offered contact information for the MSI call center or the nearest clinic if they have issues that they want to discuss further.

Taking adequate steps to protect the identity of all participants is a significant assurance, given the ethical implications that arise from the sensitive nature of the research. Protecting the privacy and confidentiality of participants will also be necessary to overcome concerns that may otherwise prevent individuals from participating in the study. In terms of data collection, data collectors (including providers and research assistants) will be trained to respect and understand how to safeguard participant confidentiality and data protection. Study participants will not be identified by name or by any other potentially identifying information (such as pharmacy name or location) in any report or publication resulting from study data. Material from web-based sources (eg, chat transcripts, photos, etc) will be gathered only with the consent of the research participants. To mitigate the risks to participants’ privacy, researchers will anonymize the source as much as possible of the material that is gathered (eg, deleting the original sound files as soon as the interview is transcribed and deleting information that might make the interviewee recognizable from the transcript itself). The potential benefits outweigh the risks: digital platforms are increasingly used to search, exchange, and circulate information, including health care information, but because of the semiclosed nature of many of these platforms, very little is publicly known about the details of how this happens.

In terms of data storage, participants’ contact information and names will be stored separately from questionnaires/transcripts on contact information sheets, which will link to the questionnaire and interview data using a unique personal ID for each participant. Contact information and informed consent documents will be stored in a locked cabinet in a locked room in the MSIC office. All the data forms/questionnaires will be kept under the custody of authorized personnel in locked cabinets. Data entry software, accessible only by study investigators, will be password protected, and full database access (also password protected) will be restricted. Data will be stored for 5 years after the project and then will be deleted. Photos will be downloaded daily from digital cameras or phones onto the computer of the researcher who has taken them. They will be subsequently deleted from the digital device and saved only in the computer on a password-protected folder. The photos will be labeled in a way to preserve people’s privacy as much as possible, for example, through the use of pseudonyms and by deleting or anonymizing with photo editing software photos that might reveal personal data (eg, screenshots of mobile phones where the phone number is visible). As with other materials gathered, participants’ contact information and names will be stored separately from questionnaires/transcripts on contact information sheets, which will link to the questionnaire and interview data using a unique personal ID for each participant. Images will be analyzed through qualitative analysis software, which will be password protected and accessible only by the study investigators. Images will be used in presentations and publications if the research participants consent. All images that are not used for those purposes will be deleted 5 years after the project, as per the data management plan. The risk of loss of confidentiality may occur if any of these procedures are inadvertently breached. All possible efforts will be made to prevent this from happening.

### Further Details of Factory Workers

Activity 1 is based on qualitative ethnographic methods. The research participants will be contacted through the factory, with which there will be a memorandum of understanding. The researchers will do their utmost to avoid any factory worker feeling pressured to participate in the research project. The first phase of the activity will be observational, and the researchers will spend time in the factory to become familiar with the environment and with potential research participants. This will give potential research participants the chance to become acquainted with the researchers and to ask questions about the research in an informal and group environment. To widen the group of participants, the researchers will also rely on personal contacts of the Cambodia-based researchers and on the networks they will create once in the field, informing potential participants of the research project and the consent procedure in the same manner as in the factory. The researchers do not foresee more than the minimal risk associated with this phase of the research. Due to this and to minimize any concern about possible breaches of confidentiality, they would like approval to use oral as well as voluntary written consent. Owing to historical and cultural reasons, requiring participants who are not well educated and are from poorer backgrounds to sign written documentation, such as consent forms, can heighten their perception of the risks should confidentiality be breached. In addition, in Cambodia, signed documents may be seen as political documents rather than statements of legal facts or contracts designed to protect the research participant. The informed consent process for activity 1 will go as follows. At the beginning of the observation process, the researchers will introduce themselves and their research and tell participants that they would like to spend time informally with them. They will make it clear that participants can leave at any time or refuse any request to chat. If researchers take photos or do recordings where individuals can be identified, they will ask for permission to do so and explain how they will use such material. The researchers will anonymize any such materials as appropriate as soon as possible. For example, if they take photos of mobile phone usage, they will download the photos to their computers and blackout or photoshop information that might make the participants recognizable, such as phone numbers, avatars, and other personal information. Before the first interview, researchers will verbally review the points detailed in the consent form (Multimedia Appendix 2) and ask for verbal permission to conduct the interview. If audiotaping or photographing, they will also verbally review the points detailed in the audio consent form and in the photo consent form (Multimedia Appendix 2). If permission is given, the researchers will ask participants if they are willing to sign a written consent form (Multimedia Appendix 2). If the participant declines to sign the written consent forms but gives verbal permission, the researcher will offer the interviewee a copy of the written consent forms for her records, complete the oral consent form (Multimedia Appendix 2), and proceed with the interview. If written permission is given, the researcher will give the interviewee a copy for her records and archive the signed consent form. The oral form is the written form read by a native speaker and recorded as a sound file, which is then distributed to potential participants (through mobile phone Bluetooth, most likely) who have the time to listen, maybe with trusted friends, to the description of the research project and think about it before accepting to participate. The sound file will also remain with them if they want to hear it again. This ensures that they have the time to process information about the project and can provide informed consent without having to rely on written forms. This is aligned with research that suggests that alternatives to written consent are preferable among certain populations, especially in developing countries [20-22]. In ethnographic research, there is no expectation of independently witnessed consent—in fact, in the case of interviews, that might be considered a breach of the privacy of the interviewee. If the interviewee agrees for the interview to be recorded, then she will be asked to repeat this agreement on tape. Again, it is important to stress that the level of risk for activities 1 and 2 is rather different and that the process for informed consent outlined for activity 1 is aligned with the expectations and conventions of ethnographic fieldwork [23].

As previously mentioned, the researchers foresee minimal risks for the research participants in this phase. Possible risks include the participants being disappointed to be befriended by researchers and then lose touch with them. To mitigate this risk, researchers involved in this phase will accept any offer to stay in touch with the participants on social media and remain friends on the web until the participants desire so. The previous experience of PIs who have worked together shows that most research participants are not particularly interested in remaining in touch with researchers but those who can often become involved informally in the research as key informants and *sounding boards* for researchers’ ideas. This could be a way to involve research participants in the project in an active way, as grassroot researchers rather than only a source of information for the researchers, and make the project more grounded in the local reality. A second possible risk is that those who participate in the research might be seen with suspicion by coworkers and other people in their environment. The researchers believe that their approach is built to mitigate these risks as much as possible, as they will enter the field site with the approval of the factory management and spend a few days just observing the environment and chatting informally to a large number of people. Once potential research participants are identified, they will discreetly be asked if they are interested in being interviewed, and the place and time of the interview will be decided by them. Finally, as with all research, there is a risk of breach of confidentiality despite the procedures in place to preserve it. Disclosure of participants’ responses outside of the research would be highly unlikely to put them at risk or to be damaging to their health, financial standing, employability, or reputation.

Payments will only be made to compensate for travel expenses and refreshments but not to represent an inducement to participate. The researchers expect interviews to take place near the factory and to be paying directly for snacks and refreshments, rather than to reimburse research participants for it. If travel expenses need to be reimbursed, the researcher will follow the guidelines set out in section *Further Details for Women Seeking Abortion Services*.

### Further Details of Private Provider Workers

Each provider or factory infirmary manager and worker will be asked to give their informed consent to participate by signing a consent form. Providers will have as much time as they need to consider and ask questions. If some workers of a private provider do not want to participate, but the manager agrees to participate, the provider will be included, but nonparticipating staff members will not be expected to approach clients about the study. If the manager does not consent for the provider to participate, no other providers will be approached in that private provider.

These are some potential risks associated with research with private providers. Participating private providers may provide medical abortion medications without prescription. The research study will undertake a no harm approach to the situation by informing participating providers about local regulations on dispensing medical abortion drugs, but participating providers who continue to provide medical abortion drug without a prescription will not be reported to the authorities unless the study team encounters situations that may pose significant risk to study participants (eg, sales of misoprostol to women with known gestational age >12 weeks or sales of products that cannot be used to induce an abortion). In such cases, the provider will be visited by a member of the MSIC team, who will explain the risk this poses to the women affected and to the providers’ own business. The clinical team member will explain that if this practice continues, the program may be compelled to alert authorities about the risk. The details of participating private providers and workers will be kept confidential. Reports will not reveal the identity of participating private providers or individual providers. There is a risk of breach of confidentiality resulting in the identity of participating providers being revealed and potentially revealing illegal practices (eg, off-label sales). An initial consultation with selected providers will take place before providers are formally approached to assess the acceptability of the research procedures and the intervention.

### Further Details for Women Seeking Abortion Services

The following recruitment and consent procedure for women seeking medical abortion is adapted from studies recently, given ethical approval to evaluate interventions to increase call center support for pharmacy-provided medical abortion being conducted by MSI in Zambia and Bangladesh. The recruitment of study participants will be as follows. Preliminary inquiries with private providers in the vicinity of garment factories suggest that the number of women seeking medical abortion can vary from a few per week to >30 per week. As it is common for there to be several private providers in the vicinity of a factory, it should be feasible to have a research assistant present in the nearby area to work with providers to recruit participants. Recruitment of study participants will involve private provider workers being trained to recruit clients into the study working together with the research team, and a memorandum of understanding will be developed and agreed by both parties. The details of this recruitment procedure are described in the following paragraph. Consenting private providers will be expected to ask clients seeking medical abortion whether they are willing to participate in the study during enrollment periods. Providers will be trained through an in-person visit to inform clients purchasing medical abortion pills about the study using a standardized script, to provide interested clients purchasing medical abortion pills (regardless of indication) with a study leaflet, to introduce interested clients to a member of the research team to hear more about the study or obtain their phone number for a research team member to call them back, and to explain that speaking to a member of the research team does not constitute enrollment and the client can just hear more about the study and then decide not to take part. To increase the understanding of the generalizability of the study and recruitment of women seeking medical abortion from providers, it is necessary to keep a record about the number of clients who purchase medical abortion pills and who agree or do not agree to take part and are not informed of the study (eg, clients who visit when the participating provider is busy or not present). The record will not contain the words medical abortion or directly refer to the names of the drugs being sold to reduce the social and business risks to providers of keeping the form in the premises.

The following payments to private provider procedures are adapted from studies recently, given ethical approval to evaluate interventions to increase call center support for pharmacy medical abortion being conducted by MSI in Zambia and Bangladesh. Consenting private providers will be expected to ask medical abortion clients whether they are willing to participate in the study during the study enrollment period. The estimate is that it will take a maximum of 10 min to inform a client about this study, time that could be spent focusing on their business. Providers are also expected to remain committed to the study over the study period, so their role in data collection needs to be reflected in the reimbursement for taking part. Each participating private provider will be compensated for the time spent recruiting clients with payments of US $25 per week. In addition, a similar payment will be considered for the use of a private room in which to conduct interviews. To ensure that providers are not under pressure to ask clients to participate in the study against their will, the reimbursement will not depend on recruitment performance. It will be explained that if none of their clients are interested in participating, this will not affect their relationship with MSIC or the amount of money they receive from the study. As a result, providers have the option to participate, take the incentive, and do not inform any customers about the study.

Providers will be asked to keep track of the number of clients requesting medical abortion at their facility. They will be asked to record the number of clients who are informed of the study and who are interested in or decline to participate. The tally sheet will not include the word abortion, and the names of the drugs sold will be coded to avoid the providers facing any social or business risks because of stigma around medical abortion. Providers will be asked to store these data in a confidential area. The tally sheet will not have any information about individual purchasers or providers.

The recruitment and interview steps are as follows. Women seeking medical abortion services from participating private providers will be invited to hear more about the study and participate in it on the day they purchase the pills. The standard medical abortion procedures are as follows. First, the client attends a private provider requesting medical abortion. Next, the provider will carry out the medical abortion provision as per their standard practices with regard to any counseling on medical abortion or any eligibility assessments they typically conduct. The research procedures are as follows. For all clients who choose medical abortion, after the client has purchased the product to initiate medical abortion, they will be introduced to the study by the provider and invited to learn more about the study. Providers will introduce clients to the study using a standard script and ask whether they would be interested in speaking to somebody from the research team, who will give them more information about the study. It will be explained that speaking to somebody from the research team does not constitute enrollment and will not affect any aspect of service provision. Loss of confidentiality is deemed a potential risk to the study participants. It is possible that women accessed medical abortion without informing their close family or friends. It is likely that the woman has kept her medical abortion pill use secret from people she lives with or is close to. Several specific measures will be taken to protect the confidentiality and anonymity of medical abortion purchasers. Providers will be trained to introduce the medical abortion pill purchaser to the study in a confidential manner (eg, to ask the purchaser to move to a quiet location to introduce the study and to avoid using the word abortion when speaking to clients about the study, particularly if there are other clients in the location). If a member of the research team is present, the provider will introduce the women to a member of the research team. If a member of the research team is not present, then the provider will ask for the woman’s phone number and explain that somebody from the research team will call them. In this case, the woman will be asked what phone number they would like to be called on and whether they have any preference regarding the time of day for the call and any other preferences regarding the return call. These details will be recorded in a recruitment log. The woman will be asked whether there is any risk of someone else answering the phone and whether it is acceptable to ask for her by name and say that the researchers are calling about a market research survey. Research assistants will be told to ensure that women are in a private place where they cannot be overheard before starting the interview. Female research assistants will be used wherever possible to prevent potential suspicion of infidelity resulting from long phone calls with study participants. Either in person or by phone, the research assistant will provide information about the study. Study respondents will be asked whether they would like to have more time to think about whether they want to participate. Respondents will be given up to 48 hours to think about whether they would like to participate and will be given the option of receiving a call back from the research assistant or calling back the research assistant themselves. If the respondent provided informed consent, the research assistant will then conduct a baseline interview and arrange a time for a day 14 interview. The interviews will be conducted by phone or at a place of the interviewee’s choice (eg, their home, MSI clinic, or provider location), according to their preference. On day 14, women will be recontacted at the agreed time for a second interview by phone. If the woman does not pick up, the research assistant will make up to 3 additional attempts to get through. The research assistant will be trained to confirm the woman’s identity and have a cover story about who they are and reason for calling to ensure that women’s privacy is protected. Research assistants will be women to reduce the risk of suspicion of infidelity. At the end of the interview, a follow-up interview date and time will be arranged for a final interview on day 30. On day 30, women will be contacted at the agreed time for a follow-up interview. The same phone procedures will be used as in the day 14 interview. The proposed recruitment steps for this study differ from those undertaken for the MSI study in Zambia, whereby women were asked by the pharmacist to send an SMS text message with a unique code word to the study team and subsequently called back by the study team to receive more information on the study. This project does not propose to either ask women to send an SMS text message with a code word because previous research has shown that the use of SMS text messaging is less common in Cambodia because of literacy and phones not supporting the Khmer script, and private providers have stated that this recruitment method will be too complex. Furthermore, this study differs from the Zambia study in that a research assistant will be based on the location during the recruitment period.

There are some potential risks associated with conducting research with women seeking medical abortion. Some questions asked to women as part of the research are sensitive and risk discomfort or distress. The clients will be reminded during the consent procedure and each interview that they are free to leave the interview at any time or skip any questions they do not want to answer. If a client is experiencing health problems during either interview, they will be referred to a Marie Stopes Cambodia clinic or the call center for medical counseling. Potential harm, such as intimate partner violence, could result as a result of messages sent by the research team intended for the participant being read or listened to by a third party. To mitigate this, explicit consent to send SMS text messages or voice messages will be sought, in addition to consent for face-to-face and phone interviews. Initial messages will not mention any terms related to abortion, contraception, or MSI and take the form of the researcher asking, “how are you?” or “if you have any problems, please call me.” The nature of subsequent messages to be tested will be informed by the needs assessment during activities 1 to 3. Any intervention content that is tested will be followed up with a phone call to obtain feedback. The research team will ask whether it is likely that the messages could have been accessed by a third party and about any harm that may have resulted from this. Participants will be offered referral information if required at the end of each interview, in case they have further questions about sexual and reproductive health, and to address the fact that they might be asked sensitive questions about intimate partner violence during the study. All participants, regardless of whether they disclose an experience of violence or not, will be offered information of organizations providing support to women who have experienced domestic violence. Interviewers will be familiar with these organizations and will be able to provide further information to participants if needed. The participant will be told that she can call the study number if she needs further information in the next 6 months and, after her final interview, will be told she can contact the MSIC call center for referral information at any time. Participants in the study will be women who are self-administering medical abortion pills, and there are medical risks of taking medical abortion medications without adequate counseling or information. However, this is a risk that women in Cambodia face every day regardless of their participation in this study, and this study aims to find ways to reduce this risk for women across the country.

Payments to participants will only be made to compensate for travel expenses, refreshments, and mobile phone costs (SMS text messages and phone calls) but not to represent an inducement to participate. Thus, those participating in interviews will be given US $3-$4 to compensate for travel and a snack *if* they have to travel to meet the research team but not if they do not have to travel. This rate is derived from MSIC experience and is consistent with compensation for reasonable costs incurred by acceptors in a donor-funded voluntary surgical contraception program that is not considered an incentive. The breakdown of the US $4 payment is as follows: (1) cost of transportation (round trip) is US $2, US $1 per client for refreshments during the procedure (this covers the cost of a basic rice/meat meal) and a US $1 contribution to a phone card (if the participant has incurred phone/SMS text messaging costs).

## Results

The project was funded by the UK-based AHRC and Medical Research Council (MRC) as part of their joint AHRC-MRC Global Public Health Global Challenge Research Fund Partnership Awards. The project began on October 1, 2018, and will end on December 31, 2020. Ethical approval was obtained from the LSHTM (April 4, 2018, LSHTM Ethics Ref: 14646), from MSI (April 5, 2018, MSI protocol number: 003-18), and by the Cambodian Ministry of Health (April 27, 2018, approval number: 094NECHR). The team conducted 67 semistructured interviews with female factory workers, women who sought medical abortion, health providers, and mobile phone providers; participant observation with factory workers and health providers; and an analysis of YouTube and Facebook to understand what kind of information is available, who creates it, and how it is used. The team is currently conducting data analysis, and findings are clustered around (1) the use of mobile phones and digital resources for health-related and medical abortion -related information, (2) the experience of medical abortion care, and (3) the development of an intervention through edutainment videos. The results from the project are expected to be published between 2020 and 2021.

## Discussion

In summary, this study aims to conduct a joint research project using qualitative methods from a multidisciplinary perspective to study health information gathering and sharing among factory workers and reproductive health issues after medical abortion in Cambodia from medical, cultural, and information-sharing practice perspectives and to design a pilot intervention delivered by mobile phones to support the reproductive health of women who work in factories around Phnom Penh. To our knowledge, this is the first study of this kind. Potential limitations are that many researchers are not based in Cambodia. However, the resulting cross-cultural interactions may result in additional insights that might otherwise not have been obtained.

We plan to disseminate findings to 3 audiences. First, academics, through academic publications and conference participation; second, practitioners, through workshops and training, one half-day workshop in each country for academics and practitioners, organized halfway through the project as a way to present early findings and get feedback from experts. The workshop in Cambodia will be organized with local partners; and third, research participants. A working hypothesis is that peer-to-peer communication is an effective way to engage women in postabortion care. If fieldwork confirms this, a training workshop for women in the target population (eg, garment factory workers) who are interested in becoming peer-to-peer counselors and for health care staff will be organized to train them further in health communication and on the best practices that emerged from the field.

## Appendix

Multimedia Appendix 1 Information for Participants.

Multimedia Appendix 2 Consent Forms.

Multimedia Appendix 3 Topic Guides.

## Abbreviations

AHRC: Arts and Humanities Research Council
IM: intervention mapping
LSHTM: London School of Hygiene and Tropical Medicine
MRC: Medical Research Council
MSI: Marie Stopes International
MSIC: Marie Stopes International Cambodia
PI: principal investigator
PSL: Partnering to Save Lives
SOAS: School of Oriental and African Studies
