# Supplementary material for: Development of an Intervention to Support the Reproductive Health of Cambodian Women Who Seek Medical Abortion: Research Protocol
Source: JMIR Res Protoc. 2020 Jul 10;9(7):e17779. doi: 10.2196/17779 (PMC7382009; doi:10.2196/17779)
Supplement: Multimedia Appendix 1 [file resprot_v9i7e17779_app1.docx]

## Appendix 1: Information for participants

The following information sheets and scripts will be translated into Khmer and piloted to test comprehensibility and adapted as necessary. Information sheets will be provided in written or audio form according to preference.

| **Information for factory workers**  We are inviting you to take part in a research study. Before you decide whether or not to participate, it is important that you know why we are doing the study and what is involved. Please read the following information carefully. If you prefer we will read it to you or you can listen to it on an audiofile.  *What is the study?*   - We are asking factory workers to help us understand their lives here in the factory, in particular as they relate to looking for information, sharing information with other people, and using mobile phones. We are also interested in understanding how women who work in factories address health issues in general, where do they go if they have health issues, who do they ask for help and advice, etcetera.   *Why are we doing the study?*   - We want to find out how women find and exchange information, especially related to healthcare, and if they use mobile phones in the process and how. This is the first phase of our research. A second phase will focus more specifically on information about reproductive health services and abortion.   *Why have I been chosen?*   - You work in a factory that is collaborating with us in this research project.   *Do I have to take part?*   - No, it is up to you to decide whether to take part.   *What will happen if I take part?*   - The researcher will conduct a short interview with you now, or arrange a more convenient time and place for an interview. We will ask you some general questions about your life here in the factory, your mobile phone use, and your healthcare needs. These will be very general questions that will help us understand better your life here, and how it is different or similar to your life back home, as well as what are your views on finding healthcare support and help, and mobile phone use. We will also ask you about your mobile phone use and, if you agree, we will ask you to show us what are some of the functions or app that you particularly like on your phone, what are those that you dislike, what are the apps you use most frequently, etc. With your permission, we will record the interview. (PHOTOGRAPHING) If you are willing, I would like to take some photographs of you. I am using a digital camera, so you can review the photos right away, and I can delete any photo you do not want me to keep. - The interview will take about 30 minutes, but we can stop at any time, or continue for longer if you want to talk to us. You don’t have to answer any question you do not want to answer.   *What will you do with the information and photos you get from this interview?*   - We hope to use some of the information that you provide in our publications and presentations about this research. But we will not (unless you give permission) quote or reference you in any way that would enable a reader to identify you. We will use photographs only with your permission. All records of this interview, including audiotapes, will be kept strictly confidential and archived by code to further protect your anonymity. - We would like to take pictures of your working and living environments, as well as your daily lives – e.g. while sitting at the cafeteria or at restaurants, while looking at your phone, etc. We will always ask participants’ permission before taking picture. The pictures will be shared among researchers to better understand research participants’ lives, and saved in a password-protected folder. Some of the pictures might be used in academic publications and in public presentations. The pictures held in the folders will be deleted 5 years after the completion of the project.   *Will you recompense me for the time this takes?*   - We will give you $3-4 USD to cover travel expenses if you have to travel to attend the interview.   *What do I have to do?*   - If you agree to take part, you can either sign this document that explains what is happening to you, or let us know verbally that you understand what this interview is about, and you agree to participate.   *What are the alternatives?*   - You do not have to take part. This will not affect in any way your work here in the factory, nor your relationship with us.   *What are the possible disadvantages in taking part?*   - The study will take some of your time. We cannot promise that your health or any other aspect of your life will benefit.   *What are the possible benefits of taking part?*   - Your views can affect the way we understand how people like you find information about healthcare.   *What will happen if I don’t want to carry on with the study?*   - You can withdraw from the study at any time by letting the researcher conducting the interview know and the data collected from you will not be used. This is a friendly chat. If you want to take a break, or do not want to answer specific questions, or want to talk about different issues, you should tell us so. We hope you will feel comfortable talking with us, and we will be happy to stop at any time.   *What if there is a problem?*   - You can discuss this with the researcher present or call us on xxxxxxx. If you want to make a complaint, please call us. If you would like to make a formal complaint write to Dr. Elisa Oreglia who will follow the complaints procedure.   *Will my taking part in this study be kept confidential?*   - Yes. Your comments will be identified by a research number only and all your comments will be made anonymous.   *What will happen to the results of the research study?*   - We will use your views to improve our understanding of how people look for healthcare information at a time when mobile phones are becoming common, and possibly influence the development of new services that are easier to use than existing ones.   *Who is organising and funding the research?*   - These interviews are being run by Dr. Elisa Oreglia. The research study is run by Dr. Chris Smith (lead investigator) and Dr. Elisa Oreglia (co-lead investigator), a collaboration between Marie Stopes International and the University of London. The UK Arts & Humanities Council is funding the study.   *Who has reviewed the study?*   - The London School of Hygiene and Tropical Medicine (LSHTM) ethics committee, Marie Stopes International Ethics Committee, Cambodia Ethics Committee   *The team contact details:*   - Tel xxxxxxxxxxxx email: xxxxx@xxxxxxxxxx, LSHTM , Keppel St, London, WC1E 7HT   We will provide contact details (including a dedicated study mobile phone number) of a local researcher in the informed consent forms as soon as this person has been identified.  Thank you for taking the time to consider taking part. If you would like further information please speak to the research present or ring the study team on xxxxxxxxxx |
| --- |

| **Information for women seeking medical abortion**  We are inviting you to take part in a research study. Before you decide whether or not to participate, it is important that you know why we are doing the study and what is involved. Please read the following information carefully. If you prefer we will read it to you or you can listen to it on an audiofile.  *What is the study?*   - We are asking service users to help us understand reproductive health issues after medical abortion and to develop an intervention to support reproductive health for women who have recently had an abortion.   *Why are we doing the study?*   - We want to find out about reproductive health needs after medical abortion and whether additional follow up care for women who have recently had an abortion can be supportive and acceptable. More generally, we are interested in understanding how you find information about healthcare.   *Why have I been chosen?*   - You have attended a service provider that provides abortion and other reproductive health services for women.   *Do I have to take part?*   - No, it is up to you to decide whether to take part.   *What will happen if I take part?*   - The researcher will conduct a short interview with you now, or arrange a more convenient time for an interview. We will ask you about your reasons for deciding to have an abortion, previous contraceptive use, your mobile phone usage, and views and suggestions on possible interventions. With your permission, we will record the interview. The interview will take about 20 minutes. - With your permission we will arrange to contact you by text message, email, phone or other message programme both at two weeks and four weeks time to ask about your experience of taking the medical abortion medications, what symptoms you experienced, current use of contraception, and intermittently by text message, email, phone or other message programme according to your preference, to ask for your feedback about any new content / messages developed after the interview.   *Will you recompense me for the time this takes?*   - We will give you $3-4 USD to cover travel expenses if you have to travel to attend the interview.   *What do I have to do?*   - If you agree to take part you will need to and sign the consent form.   *What are the alternatives?*   - You do not have to take part. It will not affect any of the services that you receive   *What are the possible disadvantages in taking part?*   - The study will take some of your time. We cannot promise that your health will benefit. There is a risk that when we contact you, someone else answers your phone or reads your messages and finds out about your participation in the study about medical abortion. However, we will take precautions to avoid this: when we call we will take steps to ensure we are speaking to you first by verifying your name. If the phone call is picked up by someone other than you, we will say we are calling to conduct a market research survey and would like to speak to you, and arrange a time to call back. We will not say we are calling from Marie Stopes Cambodia or say anything about the topic of the call until we are certain we are speaking to you. We will also check that you are in a private location and that the timing of the call is convenient for you before we proceed with the interview.   *What are the possible benefits of taking part?*   - Your views can affect the information and support service users receive.   *What will happen if I don’t want to carry on with the study?*   - You can withdraw from the study at any time by letting the researcher conducting the interview know and the data collected from you will not be used   *What if there is a problem?*   - You can discuss this with the researcher present or call us on xxxxxxx. If you want to make a complaint, please call us. If you would like to make a formal complaint write to Dr. Chris Smith who will follow the complaints procedure.   *Will my taking part in this study be kept confidential?*   - Yes. Your comments will be identified by a research number only and all your comments will be made anonymous.   *What will happen to the results of the research study?*   - We will use your views to further understand reproductive health needs after seeking medical abortion and, if appropriate, develop an intervention to support reproductive health after abortion.   *Who is organising and funding the research?*   - The study is being run by: Dr. Chris Smith (lead investigator), a collaboration between Marie Stopes International and the University of London. The UK Arts & Humanities Council is funding the study.   *Who has reviewed the study?*   - The London School of Hygiene and Tropical Medicine (LSHTM) ethics committee, Marie Stopes International Ethics Committee, Cambodia Ethics Committee   *The team contact details:*   - Tel xxxxxxxxxxxx **email:** xxxxxx@xxxxxxxxxx, LSHTM , Keppel St, London, WC1E 7HT   Thank you for taking the time to consider taking part. If you would like further information please speak to the research present or ring the study team on xxxxxxxxxx |
| --- |

| **Information Sheet for private provider managers** (to be adapted for factory infirmary managers)   - We would like to invite you and your private provider to participate in this original research project. Before you decide whether you want to take part, it is important for you to understand why the research is being done and what your participation would involve. Please listen to the following information carefully and discuss it with others if you wish. Ask us if there is anything that is not clear or if you would like more information. - When all of your questions have been answered and you feel that you understand this study, you will be asked if you wish to participate in the study, and if yes to sign an informed consent form. You will be given a signed copy to keep.   **Purpose of study and requirements**  *What is the study?*   - The purpose of the study is to find out about reproductive health needs after medical abortion and whether additional follow up care for women who have recently had an abortion can be supportive and acceptable. More generally, we are interested in understanding how women access information about healthcare.   *Why has this provider been chosen?*   - We are approaching a selection of private providers providing medical abortion drugs in Cambodia to take part in the study.   *Do I have to take part?*   - No, you do not have to agree to taking part. If you do take part you are free to withdraw from the study at any time, and you don’t have to give a reason. Deciding not to take part and withdrawing from the study at any time will not disadvantage you or your job in any way.   *What will the study involve and what do you need from me?*   - If you take part in this study, we will ask you to help us recruit clients who are seeking medical abortion services into the study. With your permission, we will also ask each of your employees to consent to participate in the study. We will train you and all other staff who are willing to participate on how to recruit clients into the study. - You will be expected to ask all medical abortion clients if they are interested in participating in a research study to find about their experiences of using the medications. If the client agrees, you will introduce the client to a member of the research team to learn more about the study. - If you choose to participate, you will also be responsible for keeping a tally sheet which indicates how many clients have purchased the products being studied, how many were told about the research and how many were interested in taking part. The research will take place intermittently over 18 months. During the study period you will receive regular visits from a member of the research team. - If you take part in the study, we will also ask you to take part in a 15 minute interview today, or at your earliest convenience, to ask you about your pharmacy’s characteristics and sales.   *What happens to the results of the research study?*   - We will use data from the study to learn more about reproductive health needs after medical abortion and whether an additional follow up intervention could provide additional support. We will share results through written reports/publications, and we will share the results with the providers who take part in the research.   *What are the risks and benefits of taking part in the study?*   - The potential benefits of taking part are that you will receive support to help you provide information to women who purchase MA from your pharmacy. You will be reimbursed $25 each week for your role of telling clients about the study to acknowledge the time you will spend on the study and you will be contributing to a study which aims to improve the health of women in Cambodia. - There are also risks associated with the study. We may terminate your involvement in the study if there is a risk that research participants (purchasers of the medications) may be harmed by the continuation of the study in this provider. If the study uncovers practices that pose high risk to clients, such as sales of medications to women with gestational age higher than 12 weeks or sale of unsafe or ineffective products, a member of Marie Stopes Cambodia will visit your provider to provide a warning, and if the practice continues after this warning, we may be compelled to alert authorities to the risk. We will not alert authorities to the practice of sales of medications without prescription. - We will take precautions to reduce the risk of people outside our research team seeing the personal information you provide us. We will never store your name and phone number together with any other personal information that you provide us. The results from the study will be reported to MSIC and may be shared with other health organisations or published in medical journals. There will be nothing in the reports that can be used to identify you. You may also experience some inconvenience from the time that you and other staff spend telling clients that they can take part in the study.   *Who is organizing the research?*   - This study is being organized by the London School of Hygiene and Tropical Medicine and Marie Stopes Cambodia.   *Who can I contact?*   - If you’d like to talk to someone about the study, or get more information, please contact: |
| --- |

| **Information sheet for private provider workers** (to be adapted for factory infirmary workers)   - We would like to invite you to participate in this original research project. Before you decide whether you want to take part, it is important for you to understand why the research is being done and what your participation would involve. Please listen to the following information carefully and discuss it with others if you wish. Ask us if there is anything that is not clear or if you would like more information. - When all of your questions have been answered and you feel that you understand this study, you will be asked if you wish to participate in the study, and if yes to sign an informed consent form. You will be given a signed copy to keep.   **Purpose of study and requirements**  *What is the study?*   - The purpose of the study is to find out about reproductive health needs after medical abortion and whether additional follow up care for women who have recently had an abortion can be supportive and acceptable. More generally, we are interested in understanding how women access information about healthcare.   *Why has this provider been chosen?*   - We are approaching all staff who work at private providers providing medical abortion drugs in Cambodia to take part in the study.   *Do I have to take part?*   - No, you do not have to agree to taking part. If you do take part you are free to withdraw from the study at any time, and you don’t have to give a reason. Deciding not to take part and withdrawing from the study at any time will not disadvantage you or your job in any way.   *What will the study involve and what do you need from me?*   - If you take part in this study, we will ask you to help us recruit clients who are seeking medical abortion services into the study. We will train you and all other staff who are willing to participate on how to recruit clients into the study. - You will be expected to ask all medical abortion clients if they are interested in participating in a research study to find about their experiences of using the medications. If the client agrees, you will introduce the client to a member of the research team to learn more about the study. - If you choose to participate, you will also be expected to fill in a tally sheet which indicates how many clients have purchased the products being studied, how many were told about the research and how many were interested in taking part. The research will take place intermittently over 18 months. During the study period you will receive regular visits from a member of the research team. - If you take part in the study, we will also ask you to take part in a 15 minute interview today, or at your earliest convenience, to ask you about your pharmacy’s characteristics and sales.   *What happens to the results of the research study?*   - We will use data from the study to learn more about reproductive health needs after medical abortion and whether an additional follow up intervention could provide additional support. We will share results through written reports/publications, and we will share the results with the providers who take part in the research.   *What are the risks and benefits of taking part in the study?*   - The potential benefits of taking part are that you will receive support to help you provide information to women who purchase MA from your pharmacy. Your pharmacy will be reimbursed $25 each week for your role of telling clients about the study to acknowledge the time you will spend on the study and you will be contributing to a study which aims to improve the health of women in Cambodia. - There are also risks associated with the study. We may terminate your involvement in the study if there is a risk that research participants (purchasers of the medications) may be harmed by the continuation of the study in this provider. If the study uncovers practices that pose high risk to clients, such as sales of medications to women with gestational age higher than 12 weeks or sale of unsafe or ineffective products, a member of Marie Stopes Cambodia will visit your provider to provide a warning, and if the practice continues after this warning, we may be compelled to alert authorities to the risk. We will not alert authorities to the practice of sales of medications without prescription. - We will take precautions to reduce the risk of people outside our research team seeing the personal information you provide us. We will never store your name and phone number together with any other personal information that you provide us. The results from the study will be reported to MSIC and may be shared with other health organisations or published in medical journals. There will be nothing in the reports that can be used to identify you. You may also experience some inconvenience from the time that you and other staff spend telling clients that they can take part in the study.   *Who is organising the research?*   - This study is being organized by the London School of Hygiene and Tropical Medicine and Marie Stopes Cambodia.   *Who can I contact?*   - If you’d like to talk to someone about the study, or get more information, please contact: |
| --- |

| **Script for providers to introduce study to women seeking medical abortion**  Are you buying these medications for yourself or for someone else?  If for themself:   - There is a study about these medications that I would like to tell you about. Marie Stopes Cambodia is conducting some research, and they would like to speak to people who use these medications to find out about their experiences using it. They are planning to interview several women using the products. They want to know more about where people get information about the product, how well it worked for them and if there is anything that could be improved, so that they can better support women using these products. If you are interested in hearing more about the study from a trained researcher, we can introduce you to a member of the research team to tell you more about the study and see whether you are interested. The study involves having three short interviews over the next month. - Do you have any questions? - Are you interested in learning more about the study from a researcher who is working on the study?   *If Yes:* Would you like me to introduce you to someone from the research team |
| --- |
