# Supplementary material for: Development of an Intervention to Support the Reproductive Health of Cambodian Women Who Seek Medical Abortion: Research Protocol
Source: JMIR Res Protoc. 2020 Jul 10;9(7):e17779. doi: 10.2196/17779 (PMC7382009; doi:10.2196/17779)
Supplement: Multimedia Appendix 2 [file resprot_v9i7e17779_app2.docx]

## Appendix 2: Consent forms

The following consent forms will be translated into Khmer and piloted to test comprehensibility and adapted as necessary. Information will be provided in written or audio form according to preference.

| **Consent for factory workers (Activity 1)**  I ______________ have read/listened to and understood the participants’ information sheet (or it has been read to me). You have explained what you are trying to find out and why you would like to talk to me:   \|  \| Check box \| \| --- \| --- \| \| - I understand what the research is about \|  \| \| - I understand what is required from me if I take part \|  \| \| - I am happy for you to record our talk and any other communication between us and understand that you won’t be using my real name \|  \| \| - I am happy for you to take photos of me, provided that you show them to me and delete them if I ask you to do so. \|  \| \| - I am happy for you to use pictures taken of me in academic publications and in public presentations \|  \| \| - I have had a chance to ask any questions you have about the study \|  \| \| - I understand that I am free to decide if I want to take part or not and that I can withdraw at any time \|  \| \| - I consent voluntarily to take part in the study \|  \|     a) Participant  Name: _________________________________________  Signature:……………………………………… Date………………  b) Researcher  I____________________________________________________(name) confirm that I have carefully explained the nature and demands of the proposed research to the participant.  Signed:……………………………………………………… Date………………..  I would now like to conduct the interview with you, it will take about 30 minutes. Is it ok for me to proceed to ask you these questions? |
| --- | --- | --- | --- | --- | --- | --- | --- | --- | --- | --- | --- | --- | --- | --- | --- | --- | --- | --- |

| **Consent form for private provider managers (to be adapted for factory infirmary managers)**  Participant’s Statement:   - I confirm that I have read and understand the information sheet for the above study and have had the opportunity to ask questions. - I understand that if I decide at any other time during the research that I no longer wish for myself or my provider to participate in this project, I can notify the researchers involved and be withdrawn from it immediately without my professional or legal rights being affected. - I consent to the processing of my personal information for the purposes of this research study. I understand that such information will be treated as strictly confidential. - I agree to take part in the above study. - I agree for my provider to take part in the above study - I consent for the Marie Stopes Cambodia research team to approach my employees regarding study participation - I agree to ask clients purchasing medical abortion drugs whether they would like to hear more about a study being conducted, and to introduce them to the research team.   a) Participant  Name: _________________________________________  Name of provider:________________________________  Signature:……………………………………… Date………………  b) Researcher  I___________________________(name) confirm that I have carefully explained the nature and demands of the proposed research to the participant.  Signed:……………………………………………………… Date……………….. |
| --- |

| **Consent form for private provider workers (to be adapted for factory infirmary workers)**  Participant’s Statement:   - I confirm that I have read and understand the information sheet for the above study and have had the opportunity to ask questions. - I understand that if I decide at any other time during the research that I no longer wish to participate in this project, I can notify the researchers involved and be withdrawn from it immediately without my professional or legal rights being affected. - I agree to take part in the above study. - I agree to ask clients purchasing medical abortion drugs whether they would like to hear more about a study being conducted, and to introduce them to the research team.   a) Participant  Name: _________________________________________  Name of pharmacy: _______________________________  Signature:……………………………………… Date………………  b) Researcher  I____________________________________________________(name) confirm that I have carefully explained the nature and demands of the proposed research to the participant.  Signed:……………………………………………………… Date……………….. |
| --- |

| **Consent for women seeking medical abortion from private providers**  I ______________ have read and understood the participants’ information sheet (or it has been read to me). You have explained what you are trying to find out and why you would like to talk to me:   - I understand what the research is about - I understand what is required from me if I take part - I am happy for you to record our talk and any other communication between us and understand that you won’t be using my real name - I am happy for you to send messages to my phone designed to support reproductive health - I am happy for you to contact me at this phone number and ask for me by name for follow up interviews - I have had a chance to ask any questions I have about the study - I understand that I am free to decide if I want to take part or not and that I can withdraw at any time - I consent voluntarily to take part in the study - If you contact this phone number and someone else answers the phone, I am happy for you to say you are trying to contact me to conduct some market research   I would prefer you to say this if someone other than myself answers your phone when we contact you:  _________________________________________________________________________________  Most appropriate to call you for a follow up interview:  Insert time: N/A N/A  a) Participant  Name: _________________________________________  Signature:……………………………………… Date………………  b) Researcher  I____________________________________________________(name) confirm that I have carefully explained the nature and demands of the proposed research to the participant.  Signed:……………………………………………………… Date………………..  I would now like to conduct the first interview with you, it will take about 20 minutes. Is it ok for me to proceed to ask you these questions? |
| --- |
