# Supplementary material for: Development of an Intervention to Support the Reproductive Health of Cambodian Women Who Seek Medical Abortion: Research Protocol
Source: JMIR Res Protoc. 2020 Jul 10;9(7):e17779. doi: 10.2196/17779 (PMC7382009; doi:10.2196/17779)
Supplement: Multimedia Appendix 3 [file resprot_v9i7e17779_app3.docx]

## Supplementary information 3: Interview protocols/topic guides

In Activity 1, semi-structured interviews with **women working in factories** will tentatively cover these themes:

1. Background information about their lives and education
   1. When did they start working in the factory; how did they find the job;
   2. where do they come from; how was their life back home; how/with whom do they keep in touch back home, do they visit often.
   3. differences and similarities with life in the factory.
   4. Plans and aspirations for the future
2. Mobile phone ownership and use
   1. When/how/where/for how much did they buy their mobile phone;
   2. What are their favorite apps/activities; how many contacts they have; how many from the factory/from back home/etc.
   3. Where do they keep their mobile phone; have they ever lost it; do other people have access to it
   4. Do they have access to/use/own other digital technologies, e.g. a laptop. Where? How do they use it?

1. Health-seeking behavior
   1. Last time they were sick, what did they do? Who did they ask for advice?
   2. Did anyone ever ask their help when they were sick? What did they do? What happened?
   3. Have they ever used their mobile to look for information about health? Example?

*Note that researchers are not asking specific questions around abortion and contraception at this point, unless these issues come up in the course of the conversation. Specific questions about these topics will be asked in the interviews in Activity 2. Also, because of the exploratory nature of this phase, and the semi-structured format of the interviews, each interview will be different from the others, and mostly led by the interviewee and her answers. The protocol above represents an example of the questions planned in London, but the final list of questions will be developed in Cambodia, after the first period of observations.*

In Activity 2, interviews with **private providers** will tentatively cover these themes:

1. MA provision
   1. How long they have been providing MA and types of drugs
   2. Approximate number of clients
   3. Information given to clients
2. Training
   1. What formal training they have been given
   2. How they keep up-to-date
   3. What support they would like
3. Post-abortion care and support
   1. Post-abortion issues encountered
   2. Suggestions for interventions
   3. Suggestions for how to recruit women to research study and intervention

In Activity 3, interviews with **women seeking medical abortion** will tentatively cover these themes:

Baseline interview

1. Background information and reasons for abortion
   1. Work and living situation;
   2. Reasons for abortion and choosing MA from this provider
   3. Previous use of contraception
   4. Plans and aspirations for the future including fertility
2. Mobile phone ownership and use
   1. When/how/where/for how much did they buy their mobile phone;
   2. What are their favorite apps/activities; how many contacts they have; how many from the factory/from back home/etc.
   3. Where do they keep their mobile phone; have they ever lost it; do other people have access to it
   4. Do they have access to/use/own other digital technologies, e.g. a laptop. Where? How do they use it?

1. Post-abortion care and support
   1. Expectations of MA process
   2. What they would do if they needed support?
   3. Last time they were sick, what did they do? Who did they ask for advice?
   4. Did anyone ever ask their help when they were sick? What did they do? What happened?
   5. Have they ever used their mobile to look for information about health? Example?
   6. Suggestions for ways of providing support

Two-week follow up interview

1. Regarding the MA
   1. Experience of drug dosing
   2. Physical issues
   3. Time off work
   4. Any additional support required
   5. Plans for contraception use

Four-week follow up interview

- Will follow up on issues raised at the two-week interview

In Activity 5, interviews with **women seeking medical abortion** will seek views on possible interventions. The research team will elicit participants’ preferences for the intervention and seek comments from them on any preliminary messages/content developed, specifically asking about the acceptability, comprehensibility and appropriateness of the messages/content developed and suggestions for improvement. In Activity 6, the research team will seek feedback on actual messages sent to participants’ phones.
